# Supplementary figures and images for: Sequence amplification via cell passaging creates spurious signals of positive adaptation in influenza virus H3N2 hemagglutinin
Source: Virus Evol. 2016 Oct 3;2(2):vew026. doi: 10.1093/ve/vew026 (PMC5049878; doi:10.1093/ve/vew026)

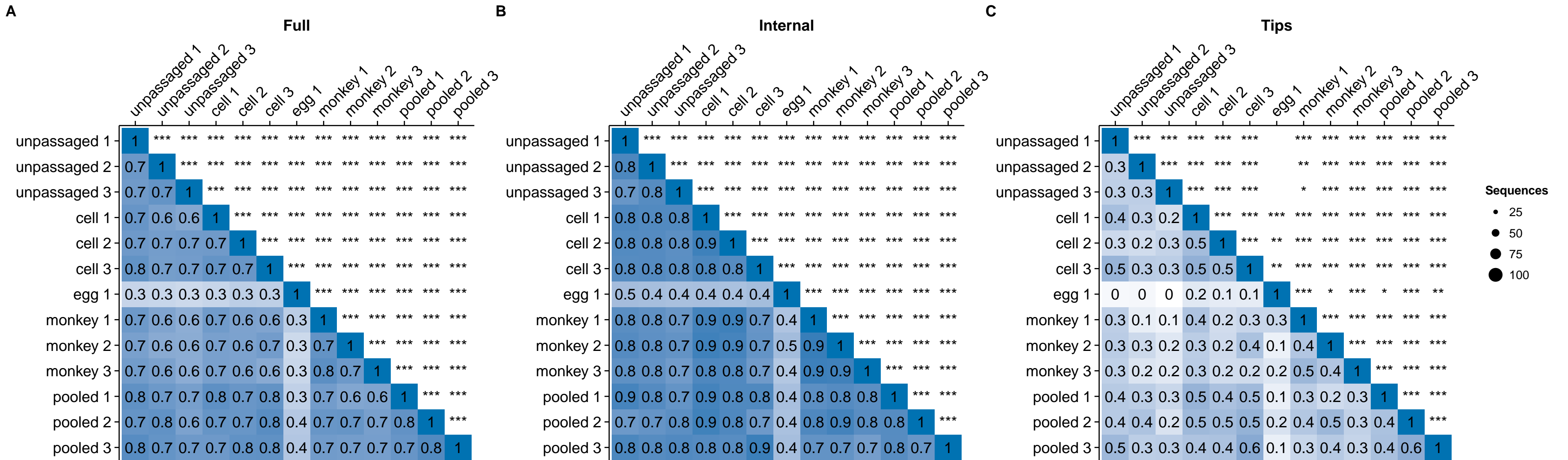

Supplement: Supplementary Data [file vew026_suppdata.zip › NIHMS813909-supplement-Figure_S1.pdf]

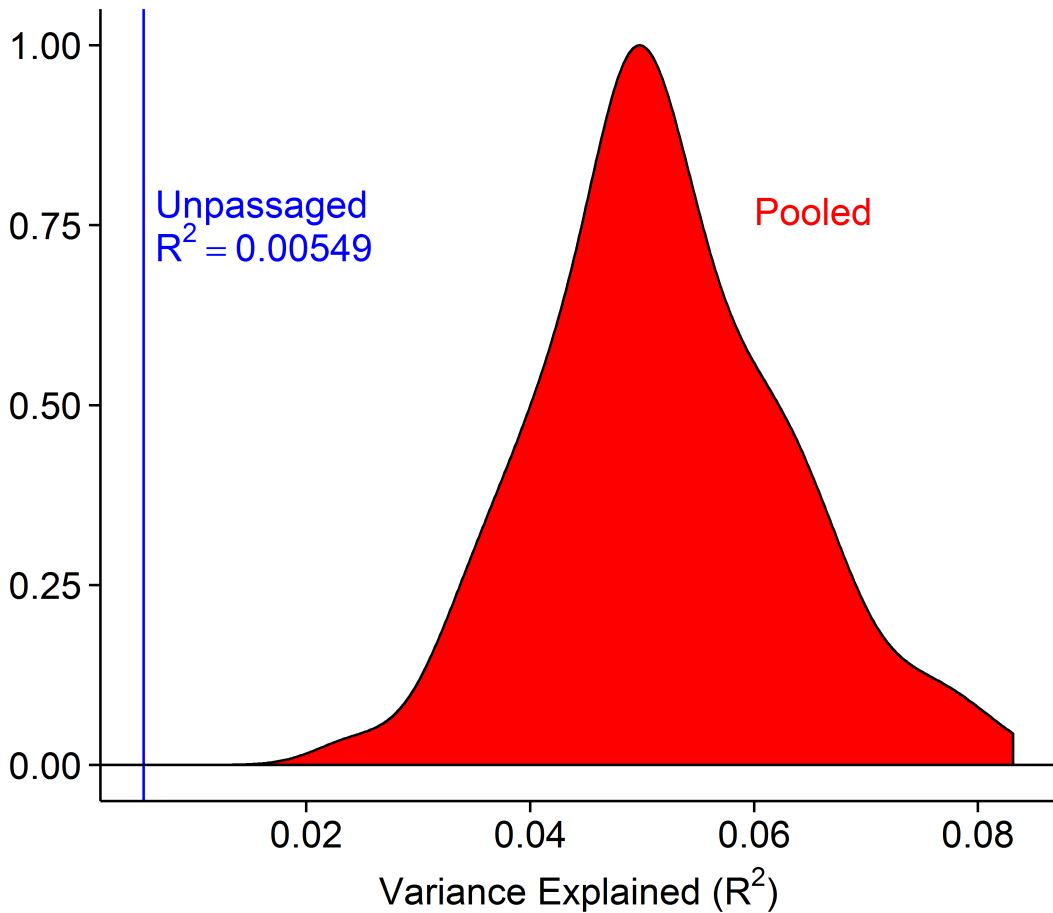

Supplement: Supplementary Data [file vew026_suppdata.zip › NIHMS813909-supplement-Figure_S2.pdf]

**A****Full**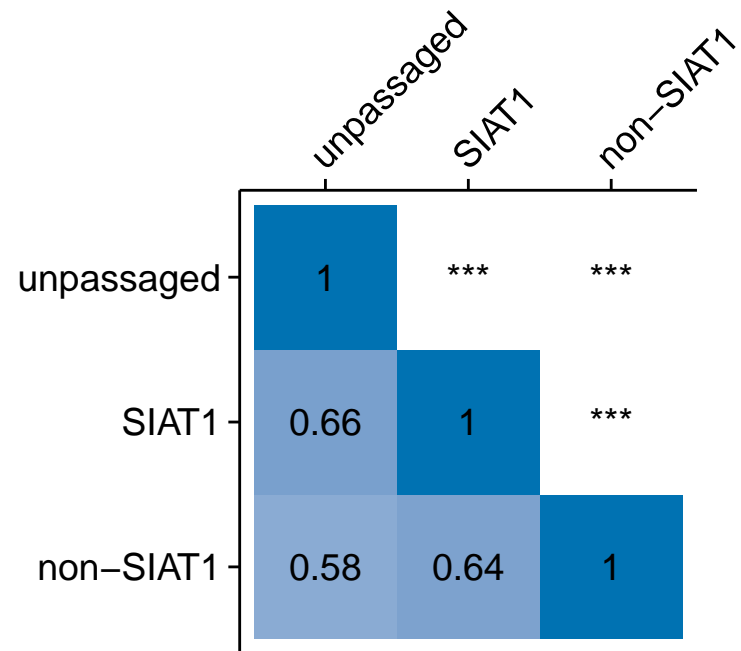**B****Internal**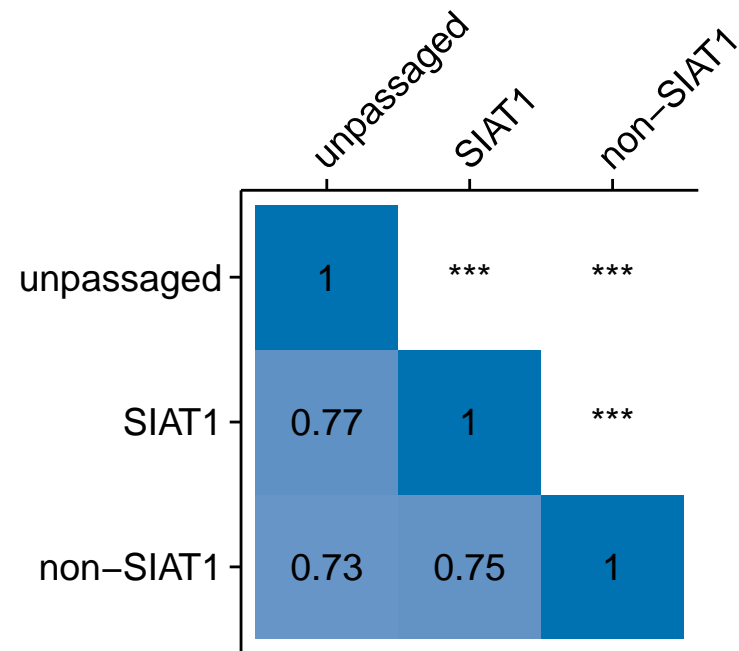**C****Tips**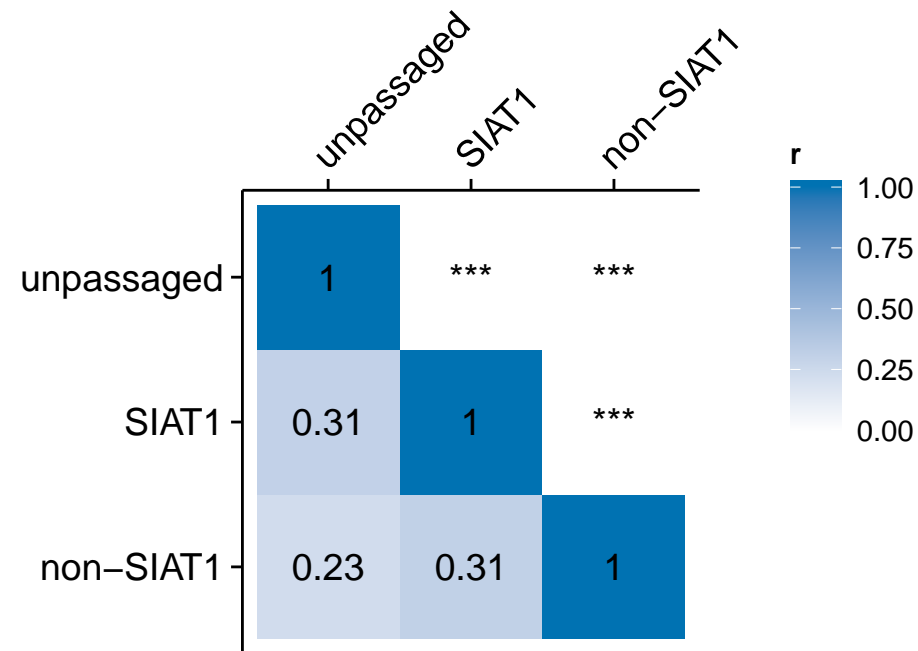

Supplement: Supplementary Data [file vew026_suppdata.zip › NIHMS813909-supplement-Figure_S3.pdf]

A

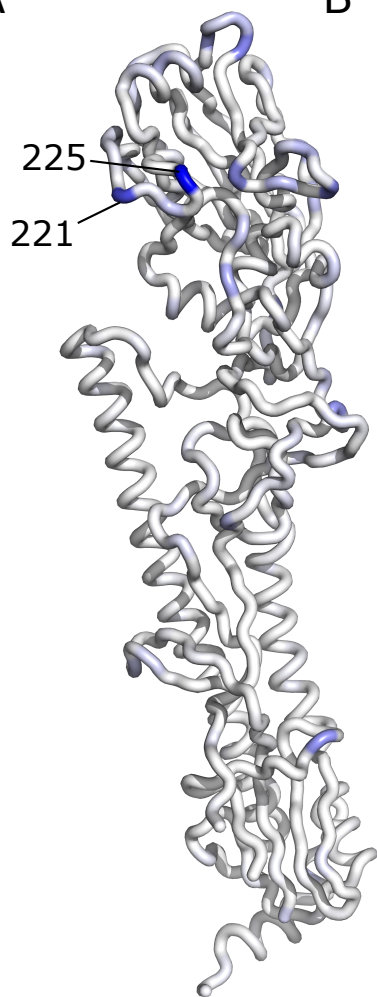

**Non-SIAT1  
passaged  
n=1046**

B

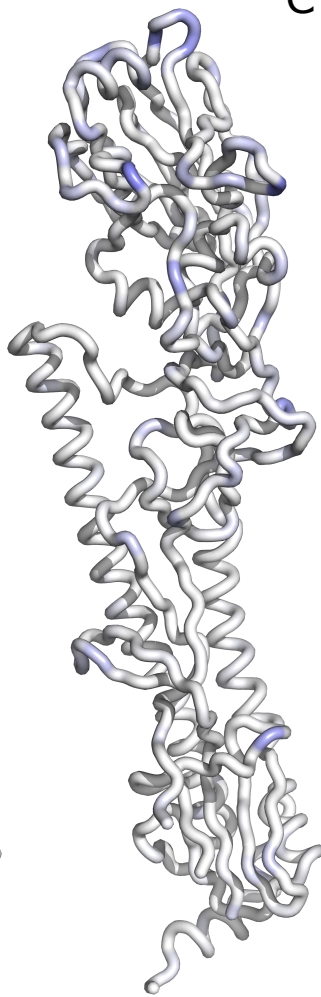

**Pooled  
sequences  
n=1046**

C

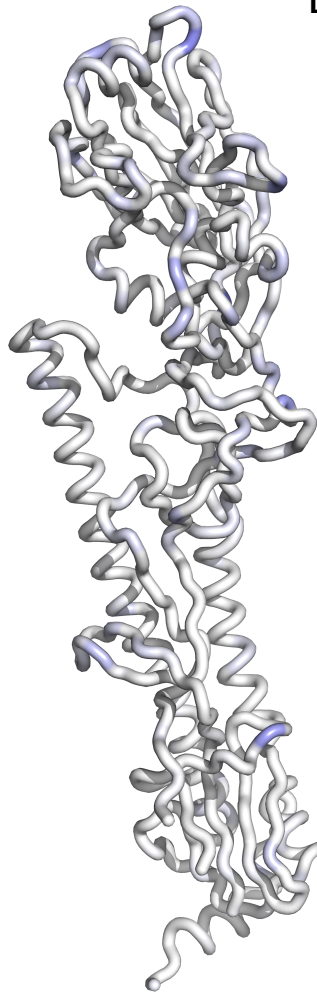

**Unpassaged  
n=1046**

D

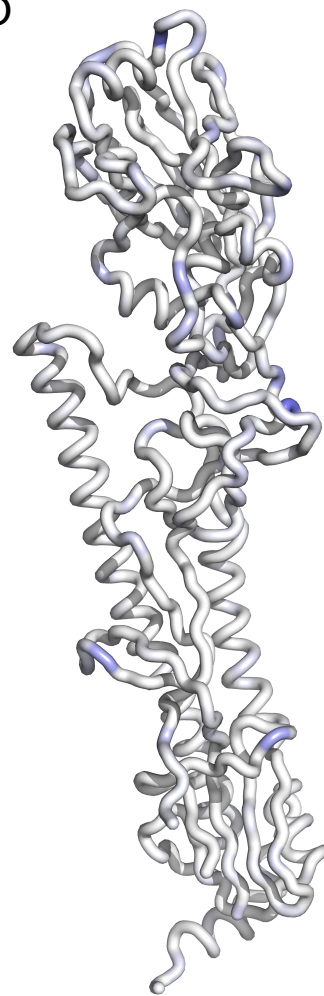

**SIAT1  
passaged  
n=1046**

0.0

 $dN/dS$ 

7.5

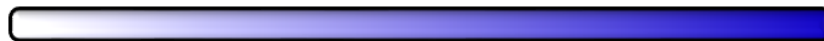

Supplement: Supplementary Data [file vew026_suppdata.zip › NIHMS813909-supplement-Figure_S4.pdf]
